# Downstream Target Analysis for miR-365 among Oral Squamous Cell Carcinomas Reveals Differential Associations with Chemoresistance

**DOI:** 10.3390/life14060741

**Published:** 2024-06-10

**Authors:** Brendon Yu, Nathaniel Kruse, Katherine M. Howard, Karl Kingsley

**Affiliations:** 1 Department of Clinical Sciences, School of Dental Medicine, University of Nevada-Las Vegas, 1700 W. Charleston Boulevard, Las Vegas, NV 89106, USA; 2 Department of Biomedical Sciences, School of Dental Medicine, University of Nevada-Las Vegas, 1001 Shadow Lane, Las Vegas, NV 89106, USA; katherine.howard@unlv.edu

**Keywords:** oral cancer, squamous cell carcinoma, chemoresistance, microRNA, downstream targets, miR-365, chemotherapy

## Abstract

Expression of microRNAs, such as miR-365, is known to be dysregulated in many tumors, including oral cancers, although little is known about their role or functions. The objective of this project is to evaluate the downstream targets of miR-365 to determine any potential pathways or effects. Downstream targets for miR-365 (miRdatabase target scores > 90) were used for qPCR screening of oral cancer cell lines (SCC4, SCC9, SCC15, SCC25, CAL27). Each oral cancer cell line expressed miR-365 downstream targets molybdenum cofactor synthesis-2 (MOCS2), erythropoietin receptor (EPOR), IQ motif containing-K (IQCK), carboxypeptidase A3 (CPA3), solute carrier family 24 member-3 (SLC24A3), and coiled-coil domain containing 47 (CCDC47)—although the expression levels varied somewhat. However, differential results were observed with ubiquitin protein ligase E3 component n-recognin-3 (UBR3), nudix hydrolase-12 (NUDT12), zinc finger CCHC-type containing-14 (ZCCHC14), and homeobox and leucine zipper encoding (HOMEZ). These data suggest that many of the miR-365 targets are expressed in the oral cancers screened, with the differential expression of UBR3, ZCCHC14, HOMEZ, and NUDT12, which may be correlated with chemoresistance among two specific oral cancer cell lines (SCC25, SCC9). These results suggest this differential expression may signal potential targets for patient treatment with tumors exhibiting miR-365 and chemotherapeutic resistance.

## 1. Introduction

Oropharyngeal cancers remain a major healthcare issue and significant healthcare burden in the United States (US) as well as other countries around the world [1,2]. Survival is most closely linked with early-stage detection and diagnosis when the tumor is less likely to have metastasized and treatment options tend to be most effective [3,4]. However, many of the oral cancers are diagnosed at late stages (particularly among elderly patients), which complicates treatment options due to regional or systemic spread that significantly reduces survival [5,6].

Surgery is among the most common methods for treating localized oral cancer to remove the primary tumor as well as any regional lymph nodes that may have been affected [7,8]. However, this treatment may also be combined with radiation or chemotherapy to eliminate other potential cancerous cells that may not have been removed through surgical resection [9,10]. New evidence suggests that radiation and chemotherapy resistance are growing concerns within the field of oral oncology treatment, and more research is focused on determining the mechanisms that mediate these phenomena [11,12]. 

Many studies now demonstrate that radiotherapy and chemotherapy resistance in oral cancers may be mediated, in part, by the expression of microRNAs within these tumors [13,14,15]. These observations are part of a growing body of evidence from other types of cancer, such as breast, ovarian, and colorectal cancers, that demonstrate the key role of microRNAs in mediating cancer development, progression, and response to treatment [16,17,18]. Based upon this information, more research has been focused in recent years on identifying specific microRNAs and their corresponding downstream targets that may function as biomarkers to determine radiation or chemotherapy resistance and sensitivity in patients [19,20].

Recent work from this group has identified miR-365 as a potential biomarker for rapidly growing and aggressive oral cancers [21,22]. Moreover, these observations with miR-365 have more recently been linked with chemotherapy resistance within these same oral cancer cell lines, suggesting that miR-365 and the corresponding downstream targets may be important mediators of oral cancer phenotypes [23,24]. However, only one study has identified a limited number of downstream targets of miR-365 in oral cancers. such as the EHF/keratin 16 and the beta5-integrin/c-met pathway, with limited or no information about any other downstream targets that could mediate oral cancer development, progression, or chemoresistance [25]. 

Similar studies of other cancers have revealed an array of microRNAs that modulate these phenotypes through specific downstream targets, such as miR-331 and the downstream target BID, as well as miR-199 and the downstream target PD-L1, that have been demonstrated to mediate both proliferation and migration among thyroid cancers [26,27]. Similarly, miR-106 has been identified as mediating progression and invasion through HPGD and Smad7, while miR-296 may function through modulation of STAT3 among esophageal squamous cell carcinomas [28,29,30]. Other research has demonstrated that some of these microRNAs may also function in specific inhibitory roles, such as miR-601, which inhibits proliferation and metastasis through specific downstream targeting of HDAC6, and miR-133, which has similar functions through the downstream targeting of the EGFR pathway, demonstrating that some microRNAs may be useful in the treatment or prevention of these cancers [31,32,33]. However, although many studies have identified the expression of microRNAs among oral squamous cell carcinomas, fewer have identified the potential downstream targets that may be involved in modulating specific phenotypes within these cancers [34,35]. 

Based on this lack of knowledge, the objective of this project is to evaluate validated and previously identified downstream targets of miR-365 (from other model systems) to determine any potential pathways or downstream targets that may be involved in oral cancer phenotypes.

## 2. Materials and Methods

### 2.1. Cell Lines and Tissue Culture

Oral squamous cell carcinoma (OSCC) cell lines were obtained from the American Type Culture Collection, or ATCC (Manassas, Virginia, USA), including SCC4 (CRL-1624), SCC9 (CRL-1629), SCC15 (CRL-1623), SCC15 (CR-1628), and CAL27 (CRL-2095). Human gingival fibroblasts, or HGF-1 cells (CRL-2014), were used as a normal, non-cancerous control. All cells were maintained in Dulbecco’s Modified Eagle’s Medium (DMEM:F12) with the addition of 10% fetal bovine serum (FBS) and 1% penicillin-streptomycin antibiotic obtained from Fisher Scientific (Fair Lawn, NJ, USA). Cells were maintained in 25 cm^2^ tissue culture-treated flasks in a biosafety level two (BSL2) cabinet with supplemental CO_2_ at 5%, as previously described [21,22,23,24]. 

Verification of each cell line was provided by ATCC using short tandem repeat (STR) analysis. The designation of each as oral squamous cell carcinoma (OSCC) was validated with scores above 90%. In addition, demographic information from each of the original samples (including the age of the donor at the time of sampling and sex) was provided as summarized information, as follows:

SCC4  CRL-1624  Age: 55 years old Sex: Male
Short tandem repeat (STR) confirmation: 92% Oral squamous cell carcinoma (OSCC)
STR analysis:
vWA: 15,17  TPOX: 8  TH01: 9.3  Penta_E: 14
Penta_D: 12  FGA: 21,22  D8S1179: 14  D7S820: 9,11
D5S818: 13  D3S1358: 18  D2S1338: 16,24  D21S11: 32.2
D19S433: 12,14  D18S51: 15  D16S539: 12  D13S317: 11,13
CSF1PO: 11  Amelogenin: X,Y

Additional characteristics: SCC4 cells have been characterized as epithelial, with positive expression of epidermal keratins (including 40 kD keratin).

SCC9  CRL-1629  Age: 25 years old Sex: Male
STR confirmation: 100% (OSCC)
STR analysis:
vWA: 17  TPOX: 9,11  TH01: 8,9  Penta_E: 11
Penta_D: 9  FGA: 20,25  D8S1179: 13  D7S820: 8
D5S818: 12  D3S1358: 15  D2S1338: 19,21  D21S11: 28
D19S433: 12,14  D18S51: 12,14  D16S539: 10,11  D13S317: 9
CSF1PO: 11  Amelogenin: X,Y

Additional characteristics: SCC9 cells demonstrated expression of epidermal keratins as well as low levels of involucrin.

SCC15  CRL-1623  Age: 55 years old  Sex: Male
STR confirmation: 95% (OSCC)
STR analysis:
vWA: 15,17  TPOX: 8  TH01: 9,9.3  Penta_E: 7,13
Penta_D: 9,13  FGA: 19,24  D8S1179: 10,13  D7S820: 10,11
D5S818: 12  D3S1358: 1  D2S1338: 16,23  D21S11: 30,31.2
D19S433: 15  D18S51: 16  D16S539: 12,15  D13S317: 9,14
CSF1PO: 10,13  Amelogenin: X,Y

Additional characteristics: SCC15 cells maintain expression of epidermal keratins (including 40 kD keratin)., as well as detectable levels of involucrin.

SCC25  CRL-1628  Age: 70 years old  Sex: Male
STR confirmation: 100% (OSCC)
STR analysis:
vWA: 17,19  TPOX: 8,12  TH01: 8  Penta_E: 14,15
Penta_D: 13  FGA: 20,24  D8S1179: 13  D7S820: 12
D5S818: 12  D3S1358: 17  D2S1338: 17,19  D21S11: 30
D19S433: 13,14  D18S51: 16  D16S539: 11,12  D13S317: 13
CSF1PO: 10  Amelogenin: X

Additional characteristics: SCC25 cells demonstrated expression of epidermal keratins as well as low levels of involucrin.

CAL27  CRL-2095  Age: 56 years old  Sex: Male
STR confirmation: 93% (OSCC)
STR analysis
vWA: 14,17  TPOX: 8  TH01: 6,9.3  Penta_E: 7
Penta_D: 9,10  FGA: 25  D8S1179: 13,15 D7S820: 10
D5S818: 11,12  D3S1358: 16  D2S1338: 23,24 D21S11: 28,29
D19S433: 14,15.2 D18S51: 13  D16S539: 11,12  D13S317: 10,11
CSF1PO: 10,12  Amelogenin: X

Additional characteristics: CAL27 cells have been characterized as epithelial, with a cytoplasm that is highly granular. Immunohistochemistry has revealed positive staining with anti-keratin antibodies. In addition, CAL27 cells were found to be resistant to treatment with CDP (cis-platinum), VDS (vindesine sulfate), and ACTD (actinomycin D).

### 2.2. Growth Assays

Cells were grown in 96-well tissue culture-treated assay plates from Fisher Scientific (Fair Lawn, NJ, USA). Cells were seeded in each well at a concentration of 1.0 × 10^5^ cells per well and allowed to grow for three days. Proliferation was measured at 24 h, 48 h, and 72 h). In addition, cells were also grown in the presence of chemotherapeutic agents, including cis-diamminedichloroplatinum or Cisplatin (NSC 119875), Paclitaxel or Taxol (NSC 125973), and 5-Fluorouracil or 5-FU (NSC 19893), sources from Selleck Chemical (Houston, TX, USA). Assays were performed using dosages within physiologic concentrations (1–10 ng/mL) observed from in vivo studies of bioavailability [36]. 

### 2.3. RNA Extraction and cDNA Synthesis

Cells were lysed and RNA extracted using the phenol-chloroform method and the TRIzol reagent from Invitrogen (Waltham, MA, USA) following the recommended protocol. In brief, cell media was removed and cells were lysed using the TRIzol reagent, which was transferred to microcentrifuge tubes with chloroform. Following centrifugation, the upper phase containing nucleic acids was transferred to a new microcentrifuge tube with the addition of isopropanol. Following centrifugation, the pellet was washed with ethanol and resuspended in nuclease-free water. RNA was converted into cDNA using the ABgene reverse iT one-step RT kit from Fisher Scientific (Fair Lawn, NJ, USA) using the manufacturer protocol, as previously described [21,22,23,24]. This reaction consisted of 1.0 ug of total RNA combined with universal random forward and reverse primers from Invitrogen (Waltham, MA, USA) and Reddy Mix RT-PCR Master Mix with RTase Blend. Each cDNA synthesis reaction was performed in a Mastercycler gradient thermal cycler using the recommended protocol of 47 °C for 30 min for reverse transcription and 72 °C for five minutes to provide final extension in a Mastercycler gradient thermal cycler from Eppendorf (Hamburg, Germany).

Amplification of microRNAs was performed with the TaqMan Advanced microRNA (miRNA) Assay conversion kit obtained from AppliedBiosystems (Waltham, MA, USA) using the manufacturer-recommended protocol, as previously described [21,22,23,24]. In brief, due to the low volume of microRNAs and the lack of poly-adenylation, this system uses a poly-adenylation reaction for use with total RNA extracted (1.0 ug) from any cell or exosome source. After the poly-adenylation was completed, a reaction for adaptor ligation was completed using the contents of the qPCR reaction plate from the previous step. Reverse transcription, or RT, reactions were then subsequently performed using the protocol and manufacturer recommendations. Finally, the amplification of cDNA derived from these reactions was used in the TaqMan miR-Amp Reaction Mix according to the manufacturer’s recommendations. RNA and cDNA were assessed using a NanoDrop 2000 spectrophotometer and absorbance readings at A260 and A280 nm to determine the quantification and quality of nucleic acids.

### 2.4. qPCR Screening

Screening was facilitated using the SYBR green qPCR master mix from Fisher Scientific (Fair Lawn, NJ, USA). Positive controls included glyceraldehyde 3-phosphate dehydrogenase (GAPDH) as the metabolic internal standard and beta-actin as the structural internal standard. The positive control for microRNA expression was miR-16. Validated downstream targets of miR-365 were selected with the MicroRNA Target Prediction Database (www.mirdb.org, accessed on 28 March 2024) using the most selective (target scores (higher than 90) that were validated previously in other model systems. Validated primer sequences for downstream targets included ubiquitin protein ligase E3 component n-recognin 3 (UBR3) target score 95, nudix hydrolase 12 (NUDT12) target score 93, molybdenum cofactor synthesis 2 (MOCS2) target score 93, erythropoietin receptor (EPOR) target score 92, zinc finger CCHC-type containing 14 (ZCCHC14) target score 92, IQ motif containing K (IQCK) target score 91, homeobox and leucine zipper encoding (HOMEZ) target score 91, carboxypeptidase A3 (CPA3 target score 91), solute carrier family 24 member 3 (SLC24A3) target score 90, and coiled-coil domain containing 47 (CCDC47) target score 90, as follows:

*Positive controls*
GAPDH forward,       5-′ATCTTCCAGGAGCGAGATCC-3′
GAPDH reverse,        5′-ACCACTGACACGTTGGCAGT-3′
Beta actin forward,       5′-GTGGGGTCCTGTGGTGTG-3′
Beta actin reverse,      5′-GAAGGGGACAGGCAGTGA-3′

*Screening primers*
miR-16 forward,        5′-TAGCAGCACGTAAATATTGGCG-3′
miR-16 reverse,       5′-TGCGTGTCGTGGAGTC-3′
miR-365 forward,      5′-ATAGGATCCTGAGGTCCCTTTCGTG-3′
miR-365 reverse,          5′-GCGAAGCTTAAAAACAGCGGAAGAGTTT-3′

*Downstream targets*
UBR3 (target score 95)
UBR3 forward,    5′-TGGCTGTTCAAGGTTTCATAGG-3′
UBR3 reverse,     5′-GGTGCCACTGCTTAGTTTTACC-3′
NUDT12 (target score 93)
NUDT12 forward,     5′-ATCCCTTGGTTACTCTAGGTGG-3′
NUDT12 reverse,      5′-GGCTGGGCCAAATAATCCTTT-3′
MOCS2 (target score 93)
MOCS2 forward,     5′-AGTGCTGAAATAACAGGAGTTCG-3′
MOCS2 reverse,      5′-TCCAAGCTCGACATATTCTTGAC-3′
EPOR (target score 92)
EPOR forward,      5′-TGGAGGACTTGGTGTGTTTCT-3′
EPOR reverse,       5′-GCAACTCTAGGGGCACGAA-3′
ZCCHC14 (target score 92)
ZCCHC14 forward,    5′-CGGACGCATTTTATGTGGAGC-3′
ZCCHC14 reverse,     5′-TCTGCGAGGACGGGATACC-3′
IQCK (target score 91)
IQCK forward,       5′-CTGCTCTACAGACTCGTCGTT-3′
IQCK reverse,        5′-AGATTCTTGCTTGACGGCTCG-3′
HOMEZ (target score 91)
HOMEZ forward,       5′-CTGGACTGCGCTATCTCTGAA-3′
HOMEZ reverse,        5′-CTGAAGGTTTTGAGCAGGTGT-3
CPA3 (target score 91)
CPA3 forward,      5′-GGGTTTGATTGCTACCACTCTT-3′
CPA3 reverse,       5′-GCCAAGTCCTTTATGATGTCTGC-3′
SLC24A3 (target score 90)
SLC24A3 forward,       5′-TTGACCTCATGGACCTCGTAG-3′
SLC24A3 reverse,        5′-GGCATCTTCGGAAGTCAGAGT-3′
CCDC47 (target score 90)
CCDC47 forward,       5′-TCTCCTCAACGGGTCATAATCA-3′
CCDC47 reverse,        5′-GGTTCACTCTCAGTATCTCCCT-3′

### 2.5. Transfection and Overexpression of miR-365

Transient overexpression was performed using RNA produced with the RNA polymerase in vitro transcription kit from Invitrogen (Waltham, MA, USA) and the miR-365-generated cDNA. Briefly, 1.0 ug of cDNA was mixed with dNTP, RNase inhibitor, depc-treated dH20, and RNA polymerase and incubated for two hours at 37 °C. The reaction was stopped using 2 uL of 0.5 M ethylenediaminetetraacetic acid (EDTA) at pH 8.0 added to each reaction and incubated at 65 °C for ten minutes. DNase treatment [1.0 ug] was added and incubated for three hours at 37 °C. Transfection of the RNA into each cell line was completed using the mammalian transfection kit from Agilent Technologies (Everett, WA, USA). In brief, each cell line was incubated with the calcium phosphate (CaPO4) reagent and RNA produced from the PCR-generated cDNA. Transfection efficiency was confirmed using the beta-galactosidase assay (79.1%). Cells were then plated in 96-well growth assays at a concentration of 1.0 × 10^5^ cells per well and allowed to grow for three days. Proliferation was measured at 24 h, 48 h, and 72 h), as described above. Plates at each time point were fixed using 10% buffered formalin and stained using Gentian violet 1% *w*/*v* from Fisher Scientific (Fair Lawn, NJ, USA). Experimental assays were evaluated for the experimental (transfected) and negative control (non-transfected) cells using a BioTek Elx808 microplate reader (BioTek Instruments; Winooski, VT, USA) using absorbance at 630 nm, as previously described [21,22].

### 2.6. Statistical Analysis

Data from the growth and proliferation assays were downloaded from the BioTek microplate reader into Microsoft Excel, Office 365 Version, from Microsoft (Redmond, WA, USA). Differences in parametric data were calculated using two-tailed Student’s *t*-tests. Pearson’s correlation, or R, was also calculated to determine the association between two variables, such as growth or proliferation and chemotherapy resistance, which would provide a number between +1 (strong positive correlation) and −1 (strong negative correlation).

## 3. Results

**Figure 1:**
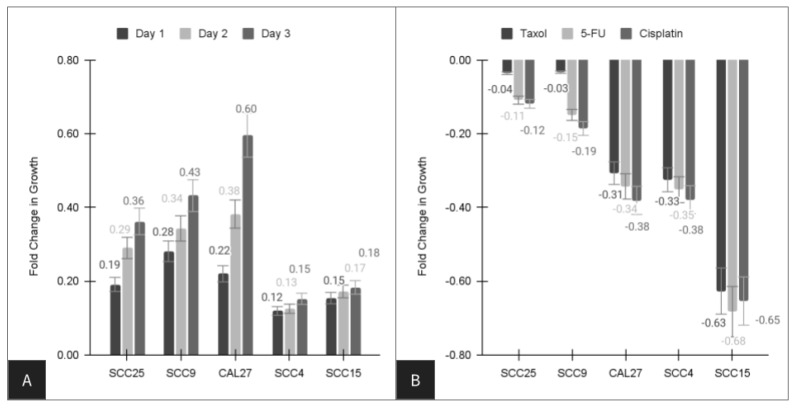
Assessment of growth among oral cancer cell lines over three days. (**A**) Growth increased in all cell lines, including SCC4 (15.2%), SCC15 (18.3%), SCC25 (36.2%), SCC9 (43.2%), and CAL27 (59.6%) cells. (**B**) Chemotherapeutic agents have differential effects among the oral cancer cell lines, such as taxol inhibiting SCC25 (−3.3%), SCC9 (−3.6%), CAL27 (−30.7%), SCC4 (−32.5%), and SCC15 (−62.7%) cells. 5-FU and Cisplatin inhibited SCC25 (−10.9%, −11.9%), SCC9 cells (−14.9%, −18.6%), CAL27 (−34.3%, −38.1%), SCC4 (−35.2%, −37.9%), and SCC15 cells (−68.3%, −65.4%) differentially.

The growth of oral cancer cell lines was assessed over three days in 96-well assays (Figure 1). More specifically, growth increased in all cell lines, with the lowest growth rates observed among SCC4 (15.2%) and SCC15 (18.3%) cells. Higher rates were observed with SCC25 (36.2%), SCC9 (43.2%), and CAL27 (59.6%) cells (Figure 1A). Administration of chemotherapeutic agents exhibited differential effects among the oral cancer cell lines (Figure 1B). For example, Paclitaxel or Taxol inhibited SCC25 (−3.3%) and SCC9 (−3.6%) only minimally, but exhibited more robust inhibition among CAL27 (−30.7%), SCC4 (−32.5%), and SCC15 (−62.7%) cells. Similarly, inhibition of oral cancers with fluorouracil, 5-FU, and cisplatin exhibited similar effects, with lower levels of inhibition among SCC25 (−10.9%, −11.9%) and SCC9 cells (−14.9%, −18.6%) and higher levels of inhibition among CAL27 (−34.3%, −38.1%), SCC4 (−35.2%, −37.9%), and SCC15 cells (−68.3%, −65.4%).

A more detailed evaluation of these results was conducted to determine the extent and range of each cell line-specific response to each of the chemotherapeutic agents (Table 1). These data demonstrated that although administration of each agent was sufficient to induce an inhibition of cell proliferation in all cell lines evaluated, these were widely variable. For example, the average inhibition of Paclitaxel or Taxol was −34.4%, but ranged from −3.3% (SCC9) and −3.6% (SCC25) to −62.7% (SCC15). Similarly, fluorouracil or 5-FU induced an average inhibition of 32.7% across all cell lines, but ranged significantly from −10.9% (SCC25) and −14.9% (SCC9) to −68.3% (SCC15). Finally, cisplatin administration reduced cell proliferation by −26.6%, ranging from −11.9% (SCC25) and −18.6% (SCC9) to −65.4% (SCC15). 

RNA was extracted from each cell line for further analysis (Table 2). This demonstrated an average RNA yield of 214.5 ng/uL, with a range between 155.4 ng/uL and 294 ng/uL. Conversion of RNA to cDNA was performed, resulting in an average cDNA concentration of 1335.6 ng/uL and a range between 1151 ng/uL and 1557 ng/uL. Analysis using A260 and A280 nm absorbances revealed an average cDNA A260:A280 ratio of 1.86, with an observed range between 1.80 and 1.92—all within the optimal range for qPCR screening and analysis. 

**Figure 2:**
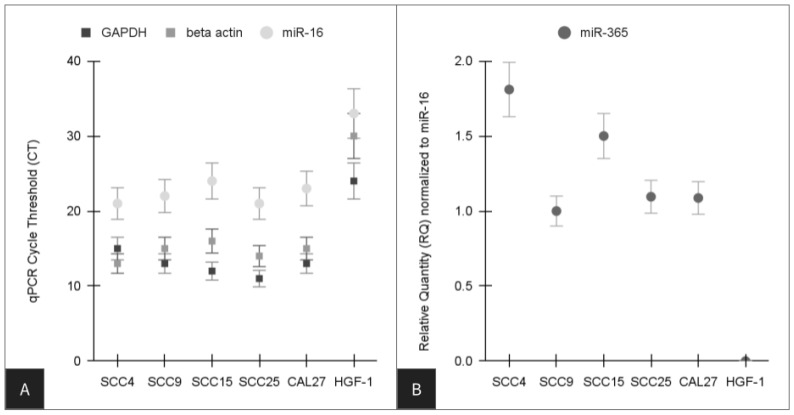
qPCR analysis of extracted RNA. (**A**) Cellular expression of the metabolic standard GAPDH (CT range: 11–24) and the structural standard beta actin (CT range: 13–30) was confirmed in all cells. (**B**) microRNA screening revealed all cells expressed the positive control miR-16 (CT range: 21 to 33), but no expression of miR-365 was detected in the normal, non-cancerous cell line, and lower expression levels and higher CT counts were observed in both SCC15 (CT: 36) and SCC4 (CT: 38) cells, which were normalized to miR-16 levels (range: 1.0 to 1.81).

To assess any differences observed among these oral cancer cell lines, the extracted cDNA was then screened using qPCR (Figure 2). Analysis of the positive controls demonstrated the metabolic standard GAPDH was expressed in all cell lines, with higher expression levels and earlier cycle thresholds (CT) observed among the oral cancers (CT range: 11–15) compared with the normal, non-cancerous control HGF-1 (CT: 24) (Figure 1A). Similarly, the structural standard beta actin was also expressed within all cells, with higher expression levels and earlier cycle thresholds (CT) observed among the oral cancers (CT range: 13–16) compared with the normal, non-cancerous control HGF-1 (CT: 30). The screening for microRNAs revealed all cells expressed the positive control miR-16 (CT range: 21 to 33). However, differences in miR-365 were observed (Figure 1B). More specifically, no expression of miR-365 was detected in the normal, non-cancerous cell line, and lower expression levels and higher CT counts were observed in the SCC15 cells (CT: 36) with the least chemoresistance, as well as the slower-growing and moderately chemoresistant cell line SCC4 (CT: 38), which were normalized to miR-16 expression levels (range: 1.0 to 1.81). 

**Figure 3:**
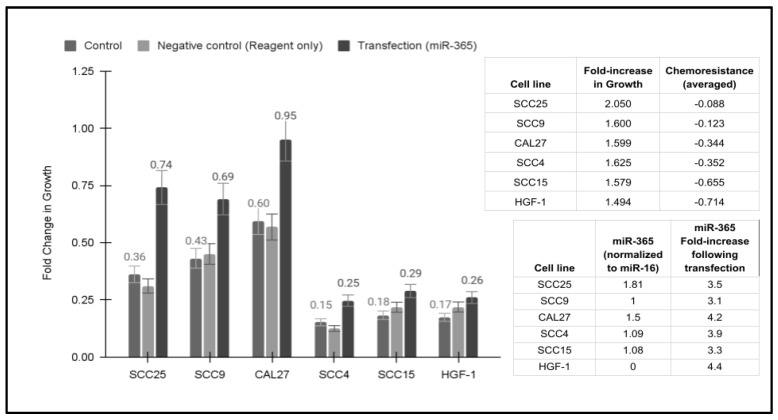
Transfection and overexpression of miR-365. Growth was increased between 1.494-fold and 2.05-fold, with the largest increase in growth observed among the most chemoresistant cell line SCC25 (average inhibition −8.8%) and the lowest increase observed among the cell lines with the least chemoresistance, including SCC15 (1.58-fold increase, average inhibition −65.5%) and the normal cell line HGF-1 (1.494-fold increase, average inhibition −71.4%), R = 0.671.

Transfection and overexpression of miR-365 revealed increased growth, with some effects among each of the cell lines evaluated (Figure 3). More specifically, the addition of exogenous miR-365 increased growth, ranging between 2.05-fold and 1.494-fold. The greatest increase in growth (2.05-fold) was observed among the most chemoresistant cell line, SCC25 (average inhibition −8.8%). Additionally, the lowest increase in cell growth with the addition of miR-365 was observed among the cell lines with the least chemoresistance, including SCC15 (1.58-fold increase, average inhibition −65.5%) and the normal cell line HGF-1 (1.494-fold increase, average inhibition −71.4%). The correlation between the fold increase in miR-365-induced growth and the average chemoresistance of each cell line was determined to be R = 0.671, suggesting a moderately positive correlation.

**Figure 4:**
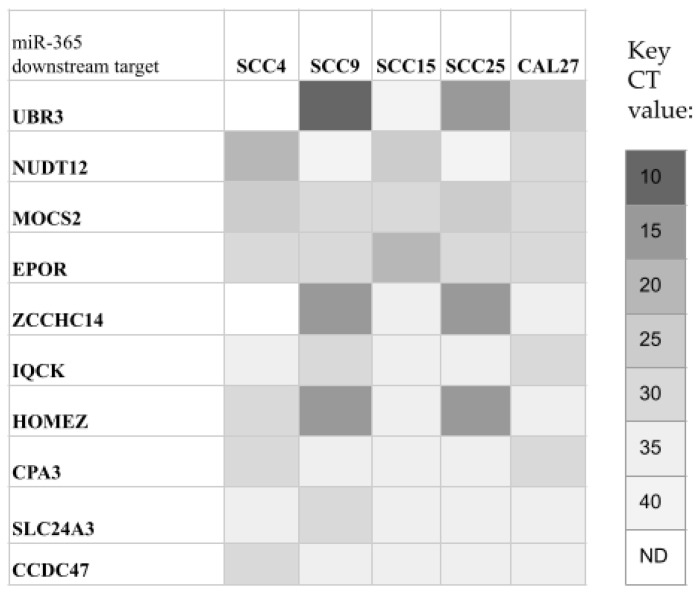
Heatmap of screening for miR-365 validated downstream targets. Oral cancer cell lines expressed CPA3, SLC24A3, IQCK, CCDC47, MOCS2, and EPOR at similar levels. Differential expression of UBR3, ZCCHC14, and HOMEZ was observed (low/no expression among SCC4 and SCC15, high expression among SCC9 and SCC25 cells). However, NUDT12 exhibited very low expression among SCC9 and SCC25 cells but moderate expression among the other oral cancer cell lines.

To further examine the potential role of miR-365, validated downstream targets were identified for screening (Figure 4). These data demonstrated all oral cancers expressed carboxypeptidase A3 (CPA3), solute carrier family 24 member 3 (SLC24A3), IQ motif containing K (IQCK), and coiled-coil domain containing 47 (CCDC47) at similar levels (CT range: 34 to 38). In addition, molybdenum cofactor synthesis 2 (MOCS2) and erythropoietin receptor (EPOR) were also expressed in all oral cancers with slightly higher expression levels (CT range: 15 to 28). 

However, more apparent differences in expression were observed among other downstream targets. For example, ubiquitin protein ligase E3 component n-recognin 3 (UBR3) and zinc finger CCHC-type containing 14 (ZCCHC14) were not expressed in SCC4 cells, and very low expression was observed among SCC15 cells (CT: 38), with relatively higher expression observed among SCC9 and SCC25 cells (CT range: 11 to 15). Similarly, homeobox and leucine zipper encoding (HOMEZ) were most highly expressed among SCC9 and SCC25 cells (CT range: 12 to 15), with lower expression observed among the other cell lines. In contrast, nudix hydrolase 12 (NUDT12) exhibited very low expression among SCC9 and SCC25 cells (CT range: 35 to 39), but moderate expression among the other oral cancer cell lines.

**Figure 5:**
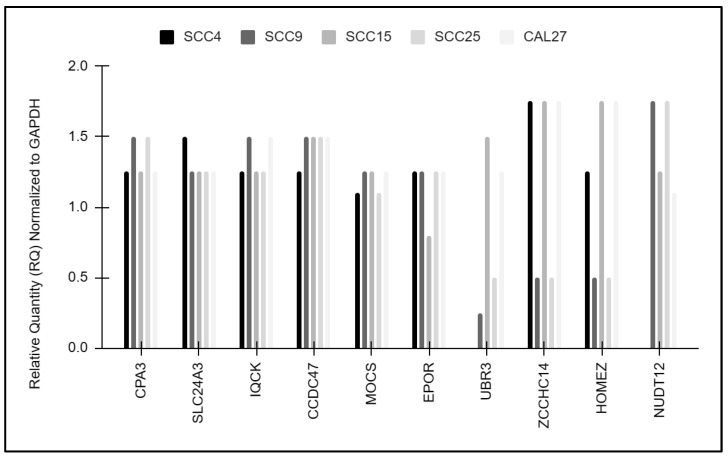
Normalization of downstream targets to GAPDH. Expression of CPA3, SLC24A3, IQCK, CCDC47, MOCS, and EPOR was consistent among the oral cancer cell lines. Variable expression of the downstream targets UBR3, ZCCHC14, and HOMEZ was observed, particularly among SCC9 and SCC25 cells, while NUDT12 expression was lower among SCC9 and SCC25 cells compared to the other cell lines.

A more detailed analysis of the results of the downstream qPCR screening was performed on each of the targets by normalizing the expression to the positive control, GAPDH (Figure 5). The data revealed that expression of CPA3, SLC24A3, IQCK, and CCDC47, as well as MOCS and EPOR, was relatively consistent among the oral cancer cell lines. However, more significant variations were apparent with the downstream targets UBR3, ZCCHC14, and HOMEZ, which were highly expressed among SCC9 and SCC25 cells but significantly lower among all other cell lines, including SCC15. In contrast, NUDT12 expression was significantly lower among SCC9 and SCC25 cells compared to the other cell lines, with the exception of SCC4, which did not express this target.

**Figure 6:**
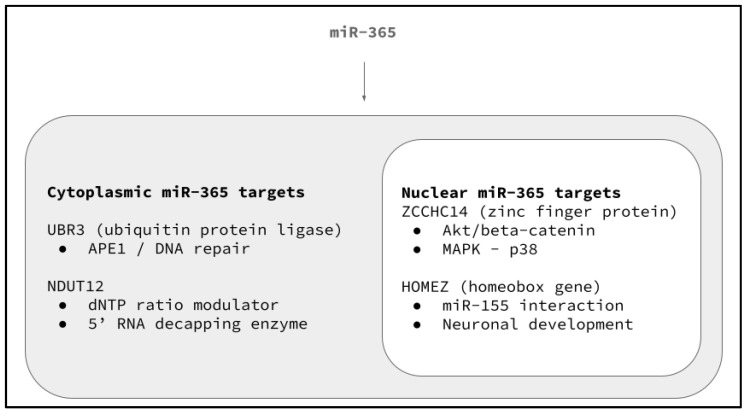
Cytoplasmic and nuclear targets for miR-365 exhibit multiple, overlapping functions. miR-365 modulates cytoplasmic UBR3 expression and translation (mediating APE1 downstream function and DNA repair), as well as NUDT12, which regulates RNA capping and decapping, as well as dNTP levels in the cytoplasm. Differential expression of miR-365 nuclear targets ZCCHC14 (mediating Akt/beta-catenin and MAPK/p38) and HOMEZ (interacting with miR-155) may also indicate differential functional relationships.

Analysis of the cytoplasmic and nuclear targets for miR-365 suggests the potential for multiple, overlapping functions (Figure 6). For example, miR-365 has been demonstrated to mediate and modulate cytoplasmic UBR3 (ubiquitin protein ligase) expression and translation, which in turn mediates APE1 downstream function and the initiation of DNA repair, which was most highly expressed among the most chemoresistant cell lines, SCC25 and SCC9. In addition, differential expression of another cytoplasmic target, NUDT12 (nudix hydrolase 12), that regulates RNA capping and decapping, as well as dNTP levels in the cytoplasm, suggests that miR-365 may also function to modulate this target within some of the cell lines. 

The expression of the miR-365 downstream target nuclear target ZCCHC14 (zinc finger CCHC-type containing 14) among SCC25 and SC9 has been demonstrated to influence further downstream targets, such as Akt/beta-catenin and MAPK/p38, which may provide a partial explanation for some of the proliferation and growth-related changes to cells following miR-365 administration. However, the expression of the potential miR-365 downstream target HOMEZ (homeobox and leucine zipper encoding) is most closely associated with neuronal development but may have additional interactions with other microRNAs that function in cancer progression and development, such as miR-155. 

## 4. Discussion

The primary goal of this project was to assess previously identified downstream targets of miR-365 to determine any potential associations with oral cancer phenotypes and to identify potential pathways of interest. This study successfully confirmed differences in phenotypes between commercially available oral cancer cell lines, with more chemoresistance observed among SCC25 and SCC9 cells and more chemosensitivity among SCC15 cells [23,24]. In addition, miR-365 expression was more highly correlated with SCC25 and SCC9 cells, as well as CAL27 cells, suggesting that pathways associated with this microRNA may be involved with these observed differences [21,22]. Moreover, analysis of miR-365 downstream targets revealed differential expression of specific UBR3, ZCCHC14, HOMEZ, and NUDT12 among the cells exhibiting the most (SCC25, SCC9) and least chemoresistance (SCC15).

More detailed analysis of ubiquitin protein ligase, or UBR3, has revealed this may be involved in regulated levels of the DNA repair protein APE1, which is considered essential for genomic repair and long-term stability [37]. Although one previous report identified UBR3 as involved with screening studies in breast cancers, this may be the first report of differential expression in oral cancers [38]. Moreover, the fact that UBR3 is most highly expressed in the most rapidly growing and most chemoresistant cell lines (SCC25, SCC9) may suggest that miR-365 modulation of this downstream target may be a significant mediator of oral cancer proliferation and progression [21,22,23,24]. 

Evaluation of zinc finger CCHC-type containing 14 or ZCCHC14 has revealed additional associations with other microRNAs, including miR-382 in gastric cancers and miR-2355 in lung carcinomas [39,40]. Moreover, previous studies have demonstrated the involvement of ZCCHC14 with Akt/beta-catenin signaling in gastrointestinal and hepatocellular carcinomas, as well as MAPK-p38 involvement in lung cancers [41,42]. As the results of this study demonstrated the highest expression of ZCCHC14 among the rapidly growing and most chemoresistant cell lines SCC25 and SCC9, more analysis of miR-365 and potential activation of these pathways may be the focus of future investigations.

The homeobox and leucine zipper-encoding protein HOMEZ has been identified as an early developmental gene in vertebrates that may function in neurogenesis [43,44]. However, more recent studies have identified HOMEZ as involved in miR-155 downstream signaling in B-cell lymphomas, which may suggest alternative pathways that involve this gene in some types of cancer [45]. The observation of differential HOMEZ expression in oral cancers, particularly among the most chemoresistant cells (SCC25 and SCC9), suggests this may be the first documentation of this phenomenon among oral cancers, and more research will be needed to determine the functional relevance of these findings. 

Finally, the results of this study also demonstrated differential expression of nudix hydrolase 12, or NUDT12, which regulates the concentrations of individual nucleotides and of nucleotide ratios in response to changing circumstances, as well as potential involvement in 5′ capping and decapping of RNA [46,47]. However, recent multi-omics screening studies have found NUDT12 may be associated with colorectal cancers as well as glioblastomas [48]. The differential expression of NUDT12 among SCC25 and SCC9 cells with high chemoresistance may suggest that either one or both of the nucleotide-regulation mechanisms associated with this protein may be involved in oral cancers, and further investigation may be warranted.

Despite the significance of these findings, this study also has limitations that should be addressed. For example, this study involves the use of commercially available oral cancer cell lines and does not address the variety and diversity of primary tumor explants. It is hoped that downstream studies and specific pathways can be identified in projects such as the current study, which would enable future researchers to validate and test these findings using patient samples [23,24]. In addition, the oral cancer cell lines from this study were all originally derived from males (mostly older), which may not necessarily influence the outcomes of this study but should also be considered when evaluating the responses and cellular behavior of these cells under chemotherapy administration. Other studies have now determined that other types of interventions, such as the use of smokeless tobacco and vaping, may have different effects and responses between males and females [49]. This may suggest that future research may need to identify and utilize oral cancers from female patients to ensure the validity and reliability of these findings. Finally, this study only utilized commercially available cell lines from the US, which may need to be significantly expanded to ensure that a more broad range of cells from other geographic regions are included to expand the diversity and range of responses observed within this study. 

## 5. Conclusions

This study confirmed that miR-365 may be a potential biomarker for rapidly growing and aggressive oral cancers that is associated with chemotherapy resistance within these same oral cancer cell lines. The results of this study further suggest that miR-365 and the corresponding downstream targets may be important mediators of oral cancer phenotypes, including UBR3, ZCCHC14, HOMEZ, and NUDT12, that are differentially expressed among the cancers exhibiting the most (SCC25, SCC9) and least chemoresistance (SCC15). Future studies that overexpress or inhibit miR-365 expression within these cancers, along with analysis of these downstream targets, mediators, and signaling pathways, may spur significant advances in the treatment of patients with chemoresistant oral cancers.

## Figures and Tables

**Table 1:** Oral cancer responses to chemotherapy administration.

Cell Line	Paclitaxel (Taxol)	Fluorouracil 5-FU	Cisplatin
SCC4	−32.5%	−35.2%	−37.9%
SCC9	−3.3% *	−14.9%	−18.6%
SCC15	−62.7%	−68.3%	−65.4%
SCC25	−3.6% *	−10.9%	−11.9%
CAL27	−30.7%	−34.3%	−38.1%
Average	−34.4%	−32.7%	−26.6%
Range	−3.3% to −62.7%	−10.9% to −68.3%	−11.9% to −65.4%

* denotes a *p*-value greater than *p* = 0.05 (not significant).

**Table 2:** RNA and cDNA analysis.

Cell Line	RNA Analysis [ng/uL]	cDNA Analysis [ng/uL]	A260:A280 Analysis
SCC4	224.3 ng/uL	1557 ng/uL	1.80
SCC9	164.4 ng/uL	1461 ng/uL	1.91
SCC15	294.4 ng/uL	1252 ng/uL	1.88
SCC25	234.2 ng/uL	1151 ng/uL	1.79
CAL27	155.4 ng/uL	1257 ng/uL	1.92
Average	214.5 ng/uL	1335.6 ng/uL	1.86
Range	155–294 ng/uL	1151–1557 ng/uL	1.80–1.92

## Data Availability

The data presented in this study are available on request from the corresponding author.
